# Towards a new strategy to implement psychosomatic knowledge in medical practice

**DOI:** 10.1186/1751-0759-2-1

**Published:** 2008-01-14

**Authors:** Hans-Christian Deter

**Affiliations:** 1 BioPsychoSocial Medicine, Universitaetsmedizin Berlin, Freie und Humboldt Universitaet, Berlin, Germany; 2 Department Psychosomatics and Psychotherapy, Charite Campus Benjamin Franklin, Universitatsmedizin Free and Humboldt University, 12200 Berlin, Germany

## Editorial

Psycho-somatic Medicine in Europe, Japan, and throughout the world must deal with similar problems such as the links between theoretical findings from different biological fields and programs in the basic sciences, and, on the other hand, progress in good clinical practice – from a psychosomatic perspective. What this means is good bio-psycho-social primary care, family and internal medicine, and detection of psychosomatic mechanisms in the development of different chronic diseases such as asthma, hypertension, coronary heart disease, inflammatory bowel disease, diabetes, atopic dermatitis and chronic arthritis.

As we better understand the mechanisms of the development in these diseases. especially the psychosocial influences, each country needs to develop a strategy to implement this knowledge into its practice of medicine.

The conditions of the health care system in Germany differ from those of France and Japan. Thus, it seems necessary to promote psychosomatic knowledge – conducting convincing psychosomatic studies in different medical fields – in the national health care system and in society. To implement this it is necessary to have cooperation between workers in government, in faculties/universities and in medical associations. However, the basis is holistic thinking about mind and body in the society. Japanese culture is in accord with holistic thinking in Europe and with the ideas of psychosomatic theory and practice in Germany. Victor von Weizsäcker and Thure von Uexküll in Germany and Yujiro Ikemi in Japan attempted to bring psychosomatic thinking into clinical practice as an advantage for all patients.

The differences between these earlier times and today in psychosomatic research are that, (1) we need good data in all medical fields to demonstrate psychosomatic interactions in different diseases, (2) we need to show that special psychosomatic strategies of treatment are useful for special psychological, biological and social targets in these diseases, and (3) to demonstrate in randomized psychosomatic clinical trials that treatment effects are comparable to other, more usual, treatments (TAU). Only in this way is it possible to bring psychosomatic experiences and knowledge into the national and international guidelines for special diseases, as have the European guidelines for prevention of coronary heart diseases (Orth-Gomer et al. 2005) [1].

This is a program in many national and international psychosomatic research centres and will be supported by the national psychosomatic societies. The communication and integration of these ongoing studies in journals such as Bio Psycho Social Medicine and in international meetings such as the World Conference on Psychosomatic Medicine (WCPM) 2005 in Kobe, where the Japanese Emperor gave support for this psychosomatic thinking, and the dissemination within the Society seems necessary. Psychosomatic networks such as the European Network on Psychosomatic Medicine [2] help combine ideas and foster actions to spread psychosomatic knowledge and bring it into the society of various nations.

The main target should be our patients, who should profit from this forthcoming of Bio Psycho Social Medicine.

## Competing interests

Hans-Christian Deter is an Editorial Board member of BioPsychoSocial Medicine
